# A Novel Demographic Indicator Fusion Network (DIFNet) for Dynamic Fusion of EEG and Demographic Indicators for Robust Depression Detection

**DOI:** 10.3390/s25216549

**Published:** 2025-10-24

**Authors:** Chaoliang Wang, Qingshu Zhou, Mengfan Li, Jiaxin Li, Jing Zhao

**Affiliations:** 1 The Key Laboratory of Intelligent Rehabilitation and Neuromodulation of Hebei Province, Department of Electrical Engineering, Yanshan University, Qinhuangdao 066004, China; wang15935859229@163.com (C.W.); v1837175290@163.com (Q.Z.); lijiaxin@stumail.ysu.edu.cn (J.L.); 2 School of Health Science and Biomedical Engineering, Hebei University of Technology, Tianjin 300132, China; mfli@hebut.edu.cn

**Keywords:** electroencephalography (EEG), demographic indicators, deep learning, feature fusion, depression

## Abstract

Electroencephalography (EEG) has proven to be effective for detecting major depressive disorder (MDD), with deep learning models further advancing its potential. However, the performance of these models may be limited by their neglect of demographic factors (e.g., age, sex, and education), which are known to influence EEG characteristics of depression. To address this, we propose DIFNet, a deep learning framework that dynamically fuses EEG features with demographic indicators (age, sex, and years of education) to enhance depression recognition accuracy. DIFNet is composed of four modules: a multiscale convolutional module, a Transformer encoder module, a temporal convolutional network (TCN) module, and a demographic indicator fusion module. The fusion model leverages convolution to process demographic vectors and integrates them with spatiotemporal EEG features, thereby embedding demographic indicators within the deep learning model for classification. Cross-validation between data trials showed that the DIFNet fusing age and years of education achieves a superior accuracy of 99.66%; the dynamic fusion mechanism improves accuracy by 0.72% compared to the baseline without fusing demographic indicators (98.94%), outperforming state-of-the-art methods (SparNet 94.37% and DBGCN 98.30%).

## 1. Introduction

Major depressive disorder (MDD) is a globally prevalent psychiatric disorder characterized by persistent low mood, loss of interest, anhedonia, and even suicidal tendencies [1]. According to a 2017 report by the World Health Organization (WHO) [2], more than 300 million people worldwide suffer from MDD. MDD has imposed a significant economic burden on both individuals and society, and effective, rapid diagnostic methods are urgently needed [3]. Currently, physician–patient communication and psychiatric questionnaire assessments are the primary diagnostic methods for depression [4]. The former depends on the physician’s expertise, while the latter may be influenced by the patient’s subjective biases, denials, and coverups. In addition, it is easy to miss and misdiagnose patients with mild depression during early screening because their clinical manifestations of low mood are very similar to those of people without depression. Therefore, many researchers have attempted to develop an objective assessment method for depression testing that does not require a complex clinical diagnosis [5].

Electroencephalography (EEG) is a brain function imaging technology with the advantages of low cost, noninvasiveness, and high temporal resolution. It has played an important role in the diagnosis of related neurological diseases, including seizures, Alzheimer’s disease, and depression [6,7]. Detecting depression using EEG involves acquiring EEG signals from the scalp and processing them to extract features associated with depression. These features such as max frequency, Renyi entropy, correlation dimension and C0 complexity have been demonstrated to be effective in depression detection [8,9,10].

In recent studies, deep learning methods have shown significant potential for application in depression detection due to their powerful feature representation and learning capabilities [7]. Thoduparambil et al. combined a CNN with a Long Short-Term Memory (LSTM) model to extract serial features of MDD with an average accuracy of 99.07% [11]. Deng et al. developed a SparNet network with an attention mechanism for extracting spatial-frequency domain features in local brain regions and the whole brain with an average accuracy of 94.37% [12]. Wang et al. utilized AlexNet to investigate the classification performance of the 128 EEG channels in MODMA [13] resting-state electroencephalogram (EEG) data, achieving 76.90% accuracy for the E40 channel [14]. Seal et al. proposed a CNN that simultaneously considers the temporal and spatial features of EEG signals with an accuracy of 99.37% [15]. Chen et al. introduced a graph pooling operation with a self-attention mechanism in the network and added global connections in the adjacency matrix based on prior knowledge, achieving an accuracy of 84.91% [16]. Li et al. developed a graph convolution network (GCN) network based on fine-grained EEG signals and graphical mutual information maximization with an accuracy of 96.37% [17]. Zhang et al. combined secondary subject partitioning and attention mechanisms with GCN to deliver an accuracy of 92.87% [18]. Liu et al. proposed an innovative depression prediction strategy that merges time–frequency complexity and electrode space topology to assist depression diagnosis with an accuracy of 98.30% [19].

Current research indicates the existence of a complex relationship between EEG characteristics and clinical manifestations of patients with depression, influenced by factors such as sex, age, and years of education [20,21,22]. These associations are potentially mediated by distinct neurobiological and cognitive pathways: sex differences may influence EEG patterns through divergent emotional regulation strategies and brain activation modes; aging is often accompanied by metabolic alterations and cognitive decline that shape brain activity; and educational attainment is thought to build cognitive reserve, thereby buffering against the clinical impact of the disease. Navarro-Bravo et al. suggested that while young people and women might initially show higher emotional intelligence (EI), these direct relationships with age and gender can change when accounting for educational level and depressive symptoms [20]. Montemurro et al. indicated that education level significantly moderates the relationship between EEG aperiodic activity and cognitive performance in aging, with a higher EEG exponent predicting poorer working memory and processing speed specifically in highly educated older adults, suggesting that the neural correlates of cognition are shaped by educational experience [21]. These complex interactions consequently highlight the necessity of integrating demographic and neurophysiological data. This approach is strongly supported by Zhang et al. and Ksibi et al., who markedly improved the performance of an EEG-based depression detection model by incorporating demographic factors [23,24]. Zhang et al. [23] proposed a custom 1D-CNN featuring an attention mechanism for the automatic fusion of demographics (e.g., age, sex), reaching 75.29% accuracy. Meanwhile, Ksibi et al. [24] utilized multiple ML models, finding that demographics fused with EEG data within a CNN architecture achieved up to 97% accuracy in a mixed-subject evaluation. However, as demographic indicators and EEG signals reside in distinct feature spaces, a key challenge remains: how to move beyond the superficial concatenation of features, as seen in prior studies, and achieve their synergistic integration.

To address this limitation, we propose a novel fusion mechanism that dynamically weights demographic indicators (age, years of education) with spatiotemporal EEG features. Distinct from existing works, DIFNet innovatively condenses demographic indicators into a dynamic scaling factor that globally calibrates the spatiotemporal EEG features, thereby achieving a more synergistic integration. The two major innovations of DIFNet are as follows:

(1) Dynamic Weighted Fusion Mechanism: This mechanism employs an interactive fusion strategy rather than simple concatenation, allowing for more nuanced integration of demographic indicators with EEG features. This approach enhances the model’s ability to capture complex relationships between different types of data.
(2) Multiscale Attention–Temporal Collaborative Architecture: This novel architecture combines a multiscale convolutional module, Transformer encoder module, and TCN module. By leveraging the strengths of each component, this architecture enables more effective feature extraction and classification, particularly in capturing long-range temporal dependencies and subtle mood-related patterns.

The remainder of this paper is organized as follows. Section 2 describes the EEG data considered. Section 3 describes the DIFNet algorithm and the procedures of performance evaluation. Section 4 presents results from 53 participants. Section 5 discusses some issues that arose from this study and outlines future work to improve upon this research. Section 6 summarizes this work.

## 2. Dataset Description

### 2.1. Dataset

In this study, we evaluated the proposed DIFNet algorithm using a mental disorder analysis dataset (MODMA), recorded by the researchers at Lanzhou University in 2020 [13]. A total of 53 subjects participated in the experiment, including 29 MDD subjects and 24 HCs (healthy controls). These subjects were categorized by professional assessment questionnaires such as the Patient Health Questionnaire-9 (PHQ-9) and Generalized Anxiety Disorder-7 (GAD-7) and underwent psychiatric assessments. During the experiment, resting-state EEG data was recorded using a Net Station device at a sampling rate of 250 Hz. A total of 128 electrodes were placed on the subjects’ scalp according to the international 10–20 system, with the reference electrode placed at Cz. The EEG data for each subject was recorded for approximately 5 min. Additionally, the MODMA dataset includes three key demographic indicators, age, sex, and years of education, providing researchers with comprehensive background data for model training and validation in studies related to neurological disorders such as depression.

Written informed consent was obtained from all participants prior to the experiment. Consent forms and study design were approved by the local Ethics Committee for Biomedical Research at the Lanzhou University Second Hospital in accordance with the Code of Ethics of the World Medical Association (Declaration of Helsinki).

### 2.2. Preprocessing

The recorded EEG data were preprocessed according to the following steps. First, considering both time performance and computational cost, 16 (Fp1/2, F3/4, C3/4, P3/4, O1/2, F7/8, T3/4, T5/6) of the 128 electrodes in the dataset were selected for further analysis [25]. Second, the selected data were processed with a notch filter at 50 Hz and a band-pass FIR filter between 1 Hz and 40 Hz. Third, the filtered data were segmented into data epochs using a 4 s time window with a 75% overlap [15]. Fourth, an automatic artifact suppression algorithm called Autoreject [26] was used to remove artifacts from the data epochs. Finally, before entering the network structure, the data underwent standardization, as indicated by Equation (1):

(1) $$z=\frac{X_{i,j}-\mu }{\sigma }$$

where $X_{i,j}$ represents the feature vector, μ represents the mean of the feature vector, and σ represents the standard deviation of the feature vector.

## 3. Methods

### 3.1. Proposed DIFNet Algorithm

**Figure 1:**
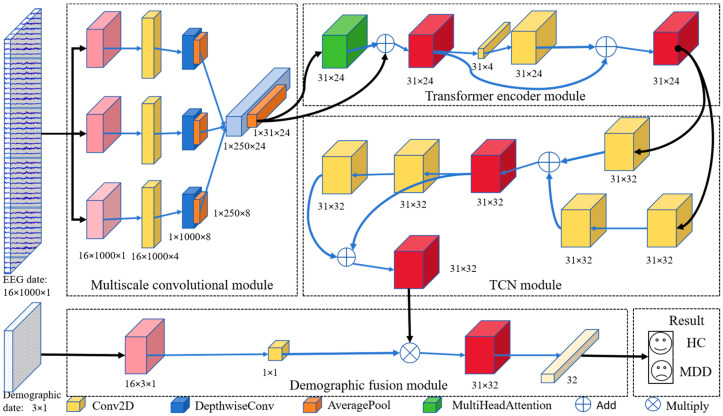
Proposed DIFNet model architecture.

The DIFNet algorithm is composed of four modules: a multiscale convolutional module, Transformer encoder module, temporal convolutional network (TCN) module, and demographic indicator fusion module (Figure 1). The multiscale convolutional module extracts spatiotemporal components of EEG signals from different frequency bands using three parallel sets of multiscale convolutional layers. The Transformer encoder module simultaneously captures the global dependencies of the feature matrix from various positions and perspectives. The TCN module extracts additional temporal features and incorporates historical information from the time series. The demographic indicator fusion module expands demographic indicator vectors using convolution and globally weights them with the spatiotemporal feature matrix for final classification.

#### 3.1.1. Multiscale Convolutional Module

**Figure 2:**
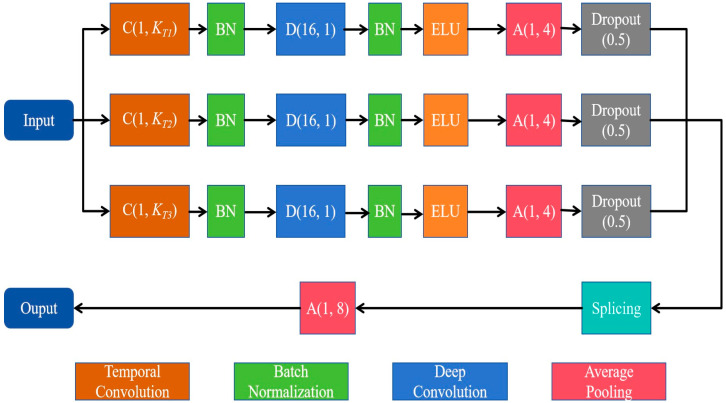
Diagram of the multiscale convolutional module.

The multiscale convolution module consists of three parallel sets of multiscale convolutional layers, each containing a temporal convolution layer for learning different rhythmic components and a deep convolution layer for extracting spatial features for each temporal filter. As shown in Figure 2, three temporal convolution kernels with different kernel sizes, C(1, *K_T_*_1_), C(1, *K_T_*_2_), and C(1, *K_T_*_3_), are used to comprehensively extract temporal features from different scales. Since the dimension of the temporal convolution kernel determines the observation window of the filter, larger kernel sizes that capture signal variations over longer time periods allow for information extraction over a wider frequency range, while smaller kernel sizes that detect signal variations over shorter time periods are suitable for separating information in higher-frequency bands. In this study, we set the kernel sizes of the three temporal convolution kernels, *K_T_*_1_, *K_T_*_2_ and *K_T_*_3_, to 250, 125 and 62, respectively.

For each parallel set, the depth convolution layer with the size of (*N_C_*, 1) then processes the signal in the space domain, with a depth multiplier of 2. Here, *N_C_* denotes the number of electrodes. Moreover, to prevent overfitting, we applied dropout (rate = 0.5) in all convolutional layers. The multiscale convolution module applies an average pooling layer of size (1, 4) and a culling layer to reduce the dimensionality of the spatiotemporal features. The output features are stitched together by a splicing layer and down-sampled by an average pooling layer of size (1, 8).

#### 3.1.2. Transformer Encoder Module

The Transformer encoder module employs a multi-head attention mechanism to learn the global dependence of EEG signals. It consists of N identical layers stacked on top of each other, each with two sublayers, the first of which is a multi-head self-attention mechanism, and the second is a positionally fully connected feedforward network. The inputs to each sublayer are connected by residuals and then normalized [27].

In deep neural networks, the attention mechanism attempts to emulate the human brain’s behavior of selectively focusing on a few significant elements while ignoring others [28]. The self-attention layer consists of three main components: query Q, keys *K*, and values *V*. Compute the dot products of the query with all keys and divide each by dk, the key of dimension. Then, apply a SoftMax function to obtain the weights of the values; the matrix of outputs is obtained using Equation (2):

(2) $$Attention(Q,K,V)=softmax(\frac{QK^{T}}{\sqrt{d_{k}}})V$$

Multi-head attention allows the model to jointly attend to information from different representation subspaces at different positions [27]. The multi-head attention layer is composed of multiple parallel self-attention mechanisms (heads). Here, the query, keys and values perform H sublinear projections by linear projection; that is, each vector is divided into H subspaces. For example, the vector Q can be expressed as $Q=\left\{X^{1},X^{2},\cdots ,X^{H}\right\}$, $Q^{H}\in {\mathbb{R}}^{N\times \frac{d_{k}}{H}}$, 1≤h≤H. The self-attention function is then performed in parallel for each subspace h, and the output of each head is concatenated and linearly transformed to obtain the raw size. The procedure can be expressed as Equation (3):

(3) $$\begin{array}{l} head^{h}=Attention(Q^{h},K^{h},V^{h})\in {\mathbb{R}}^{N\times \frac{d_{k}}{H}}\\ M(Q,K,V)=Concat(head^{1},head^{2},\cdots ,head^{H})\in {\mathbb{R}}^{N\times d_{k}} \end{array}$$

Here, *head^h^* represents the output of the h-th head after the attention mechanism; *Concat* denotes the operation used to concatenate all *head^h^*.

In this work, we incorporated two Transformer encoder modules (*N* = 2) and designed an architecture with six parallel attention heads (*h* = 6). The dimension of each attention head was set to 16 (*d_k_* = 16) to ensure the effectiveness and efficiency of the model. To prevent overfitting, we applied dropout (rate = 0.5) in the encoder module.

#### 3.1.3. Temporal Convolutional Network (TCN) Module

**Figure 3:**
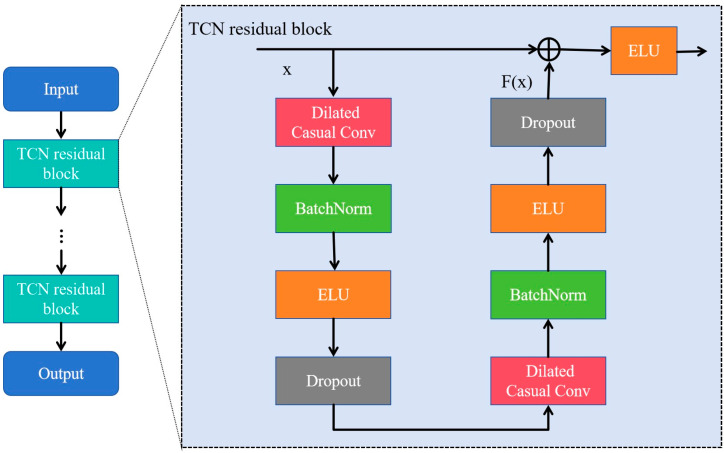
The architecture of the temporal convolutional network (TCN) consisting of multiple residual blocks. The ellipsis denotes a series of repeated blocks.

TCN is a variant of CNN commonly used in sequence-related tasks, and outperforms other recurrent architectures such as LSTM and Gated Recurrent Units (GRUs) [29,30]. Its core concept involves causal convolution, dilated convolution, and residual connections to address the inefficiencies and limited parallel computing capabilities of traditional recurrent neural networks when handling long sequence data. Moreover, it enables temporal information to be extracted from sequential data using a unique and powerful approach [31,32]. As shown in Figure 3, the TCN module consists of two TCN residual blocks that prevent gradient vanishing and explosion problems. The residual connection performs an element-wise addition of the input and output feature map, F(x)+x, which is effective in deep networks due to its ability to learn the identity function [28]. The TCN residual block is composed of two dilated causal convolution layers, each followed by a batch normalization (BatchNorm) layer and an exponential linear unit (ELU) layer.

In the TCN residual block, as the input and output dimensions are not the same, a linear transformation of 1×1 convolution is used. The receptive field size (RFS) of the TCN increases exponentially with the number of stacked residual blocks, L, due to the exponential increase in dilation D with each succeeding block. The RFS [28] is controlled by two parameters: the number of residual blocks L and the kennel size KT, as defined in Equation (4):

(4) $$RFS=1+2(K_{T}-1)(2^{L}-1)$$

TCN was selected for its capability to capture long-range temporal dependencies in EEG signals [28], which is crucial for accurately distinguishing MDD individuals from HCs. In this study, we incorporated two residual blocks (L = 2) and utilized 32 filters of size *K_T_* = 4 for all convolutional layers. The dilation factors for the dilated causal convolutions in the TCN module were set to 1 and 2, respectively. These values were empirically chosen to balance local feature extraction and global context modeling. Lastly, a dropout rate of 0.3 was applied to prevent overfitting.

#### 3.1.4. Fusion Module with Demographic Indicators

The fusion module was designed to integrate the TCN module output with the demographic indicators to improve recognition of depression patients. In this study, demographic indicators include age, sex, and years of education, and the min–max normalization is applied to them as defined in Equation (5):

(5) $$X_{norm}=\frac{X-X_{min}}{X_{\max}-X_{\min}}$$

Here, *X* represents the raw value of the particular indicator, *X_min_* denotes the minimum value in the dataset, *X_max_* denotes the maximum value in the dataset, and *X_norm_* denotes the normalized data. The normalized data of the three indicators are combined to form a demographic indicator vector *V:* $V=\left[X_{a},X_{g},X_{s}\right]$.

Demographic feature indicators and EEG features are integrated through global weighted fusion. The specific procedure is as follows: Assume *O_T_* is the output matrix of the TCN module, and its dimension is expressed as m × n. The demographic indicator vector V with the dimension of 3 × 1 is extended to 3 × n and is then convolved into a scalar feature *F*. The scalar feature *F* is fused with the output *O_T_* of the TCN module by multiplication to generate the final features for classification. A classification block with a SoftMax activation function was used in this study.

### 3.2. Performance Evaluation

Ten-fold cross-validation was performed on temporally segmented EEG trials from each subject, ensuring that trials from the same subject were distributed across training, validation, and test sets. This setup evaluates the model’s ability to generalize across temporal variations within individuals, which is critical for longitudinal monitoring of depression. To further validate the robustness of DIFNet, we conducted subject-specific finetuning experiments. For each subject, 80% of their trials were used for training and 20% for testing, and the demographic indicators in these sets were normalized separately. A total of 14,631 trials of training data were used to build a DIFNet model as described in Section 3.1. To train the DIFNet model to classify depression data, we used the Adam optimizer, known for its gradient descent efficiency, to optimize the model. In addition, we utilized the CrossEntropy loss function to measure the discrepancy between predicted and actual labels. The parameters for training the model were set as follows: the epochs were set to 200, the batch size was set to 32, and the learning rate was set to 0.001. They were determined via grid search on the validation set, optimizing for convergence speed and stability.

The DIFNet model was then adjusted using the validation set for an optimal network structure. The model parameters with the highest accuracy were saved for performance testing. For the test set, the classification accuracy, precision, recall, F1 score and kappa coefficient were calculated as shown in Equation (6).

(6) $$\begin{array}{l} Accuracy=\frac{TP+TN}{TP+TN+FN+FP}\\ Precision=\frac{TP}{TP+FP}\\ Recall=\frac{TP}{TP+FN}\\ F1=\frac{2\times Precision\times Recall}{Precision+Recall}\\ p_{e}=\frac{\left(TP+FP)\times (TP+FN)+(FN+TN)\times (FP+TN\right)}{TP+FP+FN+TN}\;\\ Kappa=\frac{Accuracy-P_{e}}{1-P_{e}} \end{array}$$

Here, *TP* denotes the true positive trials of the classification results, *TN* denotes the true negative trials, *FP* denotes the false positive trials, *FN* denotes the false negative trials, and *P_e_* denotes the expected agreement.

In this study, the hardware environment for all models was an Intel (R) Xeon Silver 4210R CPU processor (Intel, Santa Clara, CA, USA), 192 GB RAM memory, and NVIDIA GeForce RTX 3080 (10 GB) graphics card (NVIDIA, Santa Clara, CA, USA). The software environment was the Windows 10 operating system and the programming language environment was Python 3.9. Tensorflow-GPU and Keras, both version 2.60, were used.

## 4. Results

**Figure 4:**
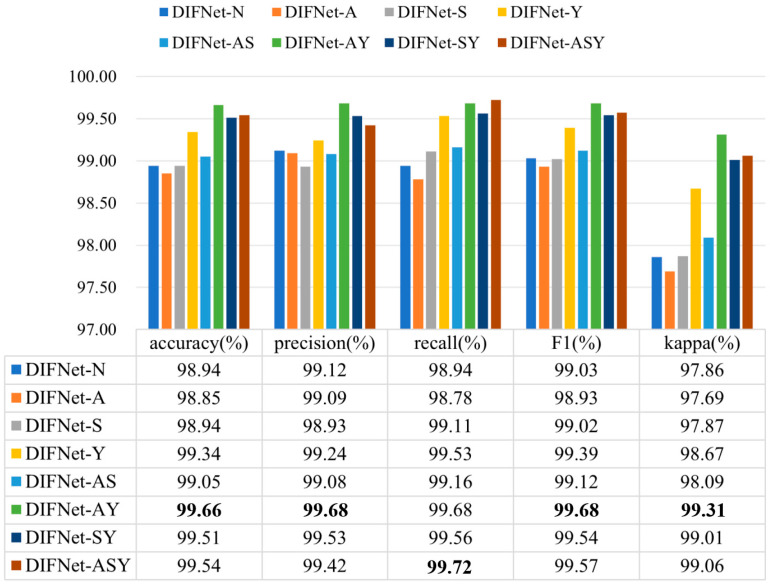
Classification results of the proposed DIFNet after fusing different demographic indicators. DIFNet-N refers to the model fusing none of the indicators, DIFNet-A refers to the model fusing age, DIFNet-S refers to the model fusing sex, DIFNet-Y refers to the model fusing years of education, DIFNet-AS refers to the model fusing both age and sex, DIFNet-AY refers to the model fusing both age and years of education, DIFNet-SY refers to the model fusing both sex and years of education, and DIFNet-ASY refers to the model fusing all three indicators. The bolded data denotes the top performance.

Figure 4 shows the classification results of the proposed DIFNet method obtained by fusing different combinations of the three demographic indicators. In the context of depression diagnosis, high accuracy indicates that the model performs well in classifying overall data. High precision means that when the model predicts that someone has depression, this prediction is likely to be correct. High recall signifies that the model can successfully identify a large proportion of actual depression cases. A high F1 score demonstrates that the model achieves both high precision and high recall, making it suitable for scenarios where both metrics need to be optimized. A high kappa coefficient suggests that the classifier’s performance significantly exceeds random guessing, especially in cases of imbalanced class distribution, where the kappa value more accurately reflects the model’s true capability.

The results showed that among these combinations, DIFNet-AY fusing both age and years of education achieved the highest average accuracy of 99.66%, highest precision of 99.68%, highest F1 score of 99.68%, and highest kappa coefficient of 99.31%, demonstrating strong within-subject consistency. DIFNet-ASY fusing age, sex, and years of education achieved the highest recall rate of 99.72% and an average accuracy of 99.54%, which is higher than that of DIFNet-N but lower than that of DIFNet-AY. Additionally, the experimental results show that compared to DIFNet-N, which did not fuse any indicators, DIFNet-Y, DIFNet-AY, DIFNet-SY, and DIFNet-ASY all demonstrated significant performance improvements (*p* < 0.05). However, DIFNet-S and DIFNet-AS showed no significant improvement (*p* = 1, *p* = 0.09). As for DIFNet-A, which fused age, although its accuracy declined, this decline was not statistically significant (*p* = 0.470); hence, we cannot conclude that the reduction in accuracy was directly caused by the fusion of age.

**Figure 5:**
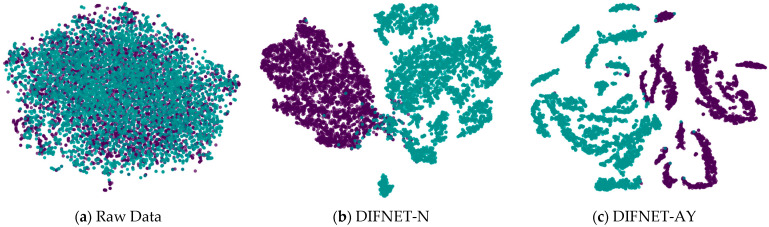
Feature representation of the representative network models. Black and purple represent HCs. Green represents MDD. HC is represented by purple, and MDD is represented by green.

Figure 5 visualizes the feature representation of the fully connected layer of representative DIFNet models for the test trials. The visualization was performed using the t-SNE algorithm [33,34]. The purple points show the feature distribution of the data trials from the HC, and the green points show those from the MDD. The results show that the proposed DIFNet method is effective in converting features mixed together in the raw data into features that can clearly distinguish between healthy controls and MDD patients. Additionally, the DIFNet model, which integrates age and education features, exhibits enhanced feature distribution and separability. The experimental results are consistent with survey studies [20,21,22,23,24], which show that higher education buffers neurodegeneration through cognitive reserve. Age-related research links specific EEG features to declines in cognitive function.

## 5. Discussion

### 5.1. Influence of Demographic Indicators on Depression Features

Demographic indicators such as age, sex, and years of education, which are related to an individual’s physical and psychological state, have been shown to affect EEG signals [23,24]. To investigate the effect of these indicators on depression-related characteristics, this study categorized the subjects according to each indicator. For example, taking into account the age indicator, subjects were categorized as under 30 years old and 30 years old and above, and differential entropy (DE), which is commonly used as a biomarker representing the degree of depression, was calculated for each category using Equation (7):

(7) $$h(x)=\int_{-\infty }^{\infty }f(x)\ln(f(x))dx$$

**Figure 6:**
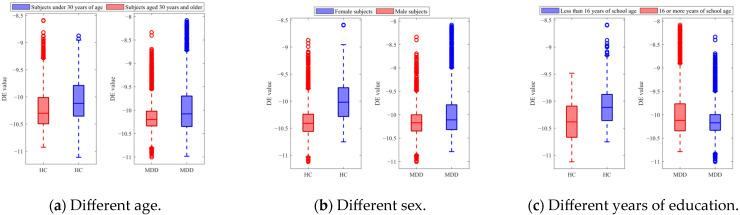
DE features of the HC and MDD subjects with different categories.

Figure 6 shows the DE features acquired from the EEG trials of the HC and MDD subjects for different categories. In Figure 6a, the red boxes represent DE values obtained from subjects under 30 years of age, and the blue boxes represent DE values obtained from subjects aged 30 years and older. The results showed that there are differences in DE features between HC and MDD subjects in different age groups. Specifically, among MDD subjects under 30 years of age, the average DE value is −10.151, with a variance of 0.096; among MDD subjects aged 30 years and older, the average DE value is −10.097, with a variance of 0.149. The DE features for healthy subjects also exhibited variation. Among HC subjects under 30 years of age, the average DE value is −10.227, with a variance of 0.148; among MDD subjects aged 30 years and older, the average DE value is −10.151, with a variance of 0.096. In Figure 6b, the red boxes represent DE values obtained from female subjects, and the blue boxes represent DE values obtained from male subjects. In Figure 6c, the red boxes represent the DE values of subjects with less than 16 years of education, and the blue boxes represent DE values of subjects with 16 or more years of education. The statistical results of paired *t*-tests showed significant differences between the DE values of both the HC and MDD subjects (*p* < 0.01) between the age categories, sex categories, and years of education categories.

**Figure 7:**
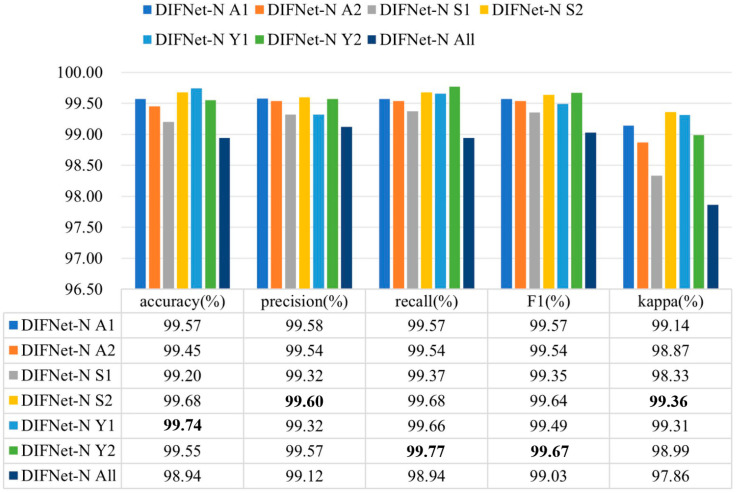
Evaluation results of DIFNet-N trained and tested using data from specific categories of subjects. The category All indicates all the subjects in the dataset, A1 indicates the subjects under 30 years of age, A2 indicates the subjects aged 30 years and older, S1 indicates the female subjects, S2 indicates the male subjects, Y1 indicates the subjects with less than 16 years of education, and Y2 indicates the subjects with 16 or more years of education. The bolded data denotes the top performance.

Figure 7 shows the evaluation results of the DIFNet-N model trained and tested using data from specific categories of subjects. Here, the category All indicates all the subjects in the dataset, A1 indicates the subjects under 30 years of age, A2 indicates the subjects aged 30 years and older, S1 indicates the female subjects, S2 indicates the male subjects, Y1 indicates the subjects with less than 16 years of education, and Y2 indicates the subjects with 16 or more years of education. The DIFNet-N method achieved higher performance in classifying the subjects of any one category than in classifying all the subjects. One possible reason is that the depression features extracted by DIFNet-N exhibit distinct distributions between different categories. As shown in Figure 7, DIFNet-N for all subjects achieved an average accuracy of 98.94%. Among other categories, DIFNet-N obtained the highest average accuracy of 99.74% in classifying data from category Y1, and the lowest accuracy of 99.20% in classifying data from category S1. Note that DIFNet-N achieved the lowest accuracy in category S1, indicating its suboptimal performance specifically for female subjects. Both Salk’s and Wang’s literature point out that depression has a higher prevalence in females and is associated with more diverse and complex psychosocial risk factors [35,36]. This higher prevalence and the variety of risk factors may lead to greater neurophysiological heterogeneity among female MDD patients, causing their EEG patterns to potentially vary more significantly from one another. Alternatively, the lack of a relatively consistent EEG pattern within the female gender itself might mean that the gender indicator contributes negatively to the classification outcome, which is also consistent with the results presented in Figure 4.

### 5.2. Influence of Frequency Band

**Figure 8:**
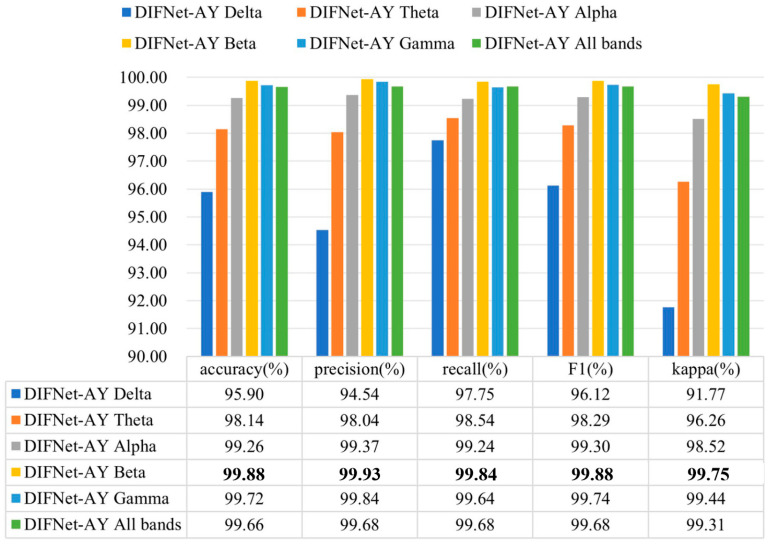
Evaluation results of DIFNet-AY trained and tested using data filtered at different frequency bands. The bolded data denotes the top performance.

Existing studies have found that the specific rhythm components of EEG signals are significantly different between MDD and HC subjects [30,31,32,33,34,37]. To evaluate the influence of different frequency bands on the proposed DIFNet algorithm, this study preprocessed the EEG data using band-pass filters for delta (1–4 Hz), theta (4–8 Hz), alpha (8–13 Hz), beta (13–30 Hz), and gamma (30–40 Hz), respectively. The filtered data for each band was then used to evaluate the performance of the DIFNet-AY model in the 10-fold cross-validation. The evaluation results of the DIFNet-AY trained and tested using filtered data from six frequency bands are shown in Figure 8.

The results showed that DIFNet-AY achieved varied performance when using different frequency bands. DIFNet-AY using the beta band achieved the highest accuracy of 99.88%, while DIFNet-AY using the delta band delivered the lowest accuracy of 95.90%. These results are consistent with the findings of Hasanzadeh et al. and Sun et al. that the alpha and beta bands contain better depression EEG information than the two low-frequency bands, delta and theta [37,38].

### 5.3. Ablation Study of DIFNet-AY

A comprehensive ablation study was conducted to evaluate the individual and synergistic contributions of the Transformer encoder (T1) and the TCN (T2) modules, as well as to validate the significance of their processing order. Table 1 shows the progressive improvement achieved by each component. The baseline model, which contains neither T1 nor T2, achieved an accuracy of 88.37%, setting a foundational performance level. Introducing the Transformer encoder alone (+T1) led to a substantial performance jump, increasing the accuracy to 98.67%. This indicates the module’s powerful capability to capture global contextual dependencies within the data. Similarly, employing the TCN alone (+T2) also resulted in a significant gain, yielding an accuracy of 99.03%, which highlights its effectiveness in modeling local temporal patterns.

The experiment with different module orders provides the most critical insight. The model +T2+T1, which processes data with the TCN followed by the Transformer, already shows excellent performance with an accuracy of 99.56%. However, our proposed configuration, +T1+T2, which first leverages the Transformer for global context and then refines features with the TCN for local precision, achieves the best overall results. It attains the highest scores across all metrics, including 99.66% accuracy and a 99.31% kappa value, thereby establishing the optimal processing sequence. In addition, the *p*-values reported in the table quantify the statistical significance of the performance difference between each model variant and our final proposed model (+T1+T2). The highly significant results (*p* < 0.001) for the baseline, +T1, and +T2 confirm that the superior performance of +T1+T2 over these models is not due to random chance. The non-significant *p*-value (*p* = 0.36) for the +T2+T1 model indicates that while our method achieves the highest scores, the difference from this particular variant is not statistically significant in this evaluation.

### 5.4. Comparison with State-of-the-Art Algorithms

The performance of the DIFNet-AY algorithm in recognizing MDD is compared with five state-of-the-art algorithms demonstrated to be effective in depression classification [12,14,18,19,24]. Table 2 shows the comparison results of these algorithms validated on the same MODMA dataset. The evaluation metrics not reported in the corresponding references are not listed in this table.

As highlighted in the Introduction, individual variability in EEG signals poses a major challenge for depression detection. The dynamic fusion mechanism in DIFNet-AY directly addresses this by adaptively weighting demographic indicators, leading to a 99.66% accuracy that surpasses SparNet (94.37%) and DBGCN (98.30%). The results show that the proposed algorithm obtained superior performance to the state-of-the-art algorithms.

### 5.5. Validation on a Reduced-Montage Dataset

To further evaluate the generalizability of our proposed method, we validated it on a public EEG dataset for MDD provided by Mumtaz et al. [39]. This dataset comprises EEG recordings from 34 MDD patients (17 female/17 male, mean age 40.3 ± 12.9 years) and 30 age-matched HCs (9 female/21 male, mean age 38.3 ± 15.6 years). All patient diagnoses conformed to the DSM-IV criteria, and this study was approved by the ethics committee of Hospital Universiti Sains Malaysia (HUSM). The EEG signals were recorded from 19 electrodes positioned over the frontal (Fp1, Fp2, F3, F4, F7, F8, and Fz), temporal (T3, T4, T5, and T6), parietal (P3, P4, and Pz), occipital (O1 and O2), and central (C3, C4, and Cz) regions.

For the validation, we first preprocessed all EEG trials from the 19 electrodes by following the preprocessing steps outlined previously. Subsequently, we shuffled the data trials from all the subjects and performed a cross-validation between these trials to evaluate the model’s performance. Since years of education are not available in the Mumtaz dataset, Table 3 consequently compares only the performance of DIFNet-A, which integrates the age indicator, against DIFNet-N, which uses no demographic indicators. The results show that the proposed DIFNet-A method achieved improved performance compared to DIFNet-N on the Mumtaz dataset with a reduced montage. This enhancement is crucial for practical use, as relying on fewer EEG channels enables the development of simpler, lower-cost, and more portable diagnostic devices.

## 6. Conclusions

This paper proposes a novel DIFNet algorithm to integrate EEG data with subjects’ demographic information to enhance the performance of recognizing MDD. The evaluation results of cross-validation between data trials showed that DIFNet, fusing age and years of education, achieved an accuracy of 99.66%, an improvement over the state-of-the-art algorithms. The higher accuracy with age and years of education fusion may stem from age-related EEG variability and education-level impacts on cognitive reserve. These results demonstrate the effectiveness of the proposed algorithm in depression classification and emphasize the importance of considering demographic factors. Despite its high accuracy, deploying DIFNet in real-world clinics necessitates addressing dataset bias (e.g., limited sample size) and ethical concerns (e.g., demographic privacy). In future work, we will consider incorporating additional demographic indicators and exploring dynamic fusion strategies to adaptively weight these indicators (such as race and socioeconomic status) based on individual EEG signatures. Furthermore, we will validate the algorithm on a broader range of datasets and validate DIFNet in multi-center trials.

## Figures and Tables

**Table 1:** Ablation results on different model variants.

Model Variants	Accuracy (%)	Precision (%)	Recall (%)	F1 (%)	Kappa (%)	*p*
Baseline	88.37	86.10	93.56	89.67	76.43	<0.001
+T1	98.67	98.74	98.81	98.77	97.33	<0.001
+T2	99.03	98.68	99.53	99.10	98.05	<0.001
+T2+T1	99.56	99.66	99.53	99.59	99.12	0.36
+T1+T2(Ours)	99.66	99.68	99.68	99.68	99.31	-

Note: T1 denotes the Transformer encoder module; T2 denotes the temporal convolutional network (TCN) module. The notation “+T1+T2” indicates that the input is processed by T1 first, followed by T2. The *p*-value is calculated from a statistical significance test (e.g., *t*-test) to compare the performance of each model against the proposed final model (+T1+T2).

**Table 2:** Experimental results of the different methods.

Scholar	Year	Model	Accuracy (%)	Precision (%)	Recall (%)	F1 (%)	Kappa (%)
Zhang et al. [23]	2020	1DCNN	75.29	-	-	71.6	-
Deng et al. [12]	2022	SparNet	94.37	-	-	94.40	-
Wang et al. [14]	2023	AlexNet	73.90	-	-	-	-
Zhang et al. [18]	2024	SSPA-GCN	92.87	92.23	92.00	91.12	-
Liu et al. [19]	2024	DBGCN	98.30	-	-	-	-
Ours		DIFNet-AY	99.66	99.68	99.68	99.68	99.31

**Table 3:** Validation results on the Mumtaz dataset with a reduced montage.

Model	Accuracy (%)	Precision (%)	Recall (%)	F1 (%)	Kappa (%)
DIFNet-N	99.87	99.83	99.91	99.87	99.74
DIFNet-A	99.93	99. 89	99.96	99.92	99.85

## Data Availability

The raw data supporting the conclusions of this article will be made available by the authors on request.

## Abbreviations

The following abbreviations are used in this manuscript:

MDD	Major Depressive Disorder
WHO	World Health Organization
EEG	Electroencephalographic
LSTM	Long Short-Term Memory
GCN	Graph Convolution Network
GRUs	Gated Recurrent Units
BatchNorm	Batch Normalization
ELU	Exponential Linear Unit
DE	Differential Entropy
TCN	Temporal Convolutional Network

## References

[1] Le G.H., Wong S., Lu A., Vasudeva S., Gill H., Badulescu S., Portelles D.R., Zheng Y.J., Teopiz K.M., Meshkat S. (2025). Electroencephalography (EEG) spectral signatures of selective serotonin reuptake inhibitors (SSRIs), selective norepinephrine reuptake inhibitors (SNRIs) and vortioxetine in major depressive disorder: A systematic review. J. Affect. Disord..

[2] World Health Organization (2017). Depression and Other Common Mental Disorders: Global Health Estimates.

[3] Greenberg P.E., Fournier A.-A., Sisitsky T., Pike C.T., Kessler R.C. (2015). The economic burden of adults with major depressive disorder in the United States (2005 and 2010). J. Clin. Psychiatry.

[4] Chen X., Pan Z. (2021). A convenient and low-cost model of depression screening and early warning based on voice data using for public mental health. Int. J. Environ. Res. Public Health.

[5] Rehman H., Khan D.M., Amanullah H., Kamran L., Rehman O.U., Siddiqui S.T., Masroor K. (2025). Advances in EEG-based detection of Major Depressive Disorder using shallow and deep learning techniques: A systematic review. Comput. Biol. Med..

[6] Zhao J., Li J., Wang X., Zhang Q., Li Z., Liang Z. (2024). Discriminating brainwave patterns of different control and non-control states for enhancing asynchronous brain-computer interfaces. Expert Syst. Appl..

[7] Li C., Li H., Dong X., Zhong X., Cui H., Ji D., He L., Liu G., Zhou W. (2025). CNN-Informer: A hybrid deep learning model for seizure detection on long-term EEG. Neural Netw..

[8] Cai H., Han J., Chen Y., Sha X., Wang Z., Hu B., Yang J., Feng L., Ding Z., Chen Y. (2018). A pervasive approach to EEG-based depression detection. Complexity.

[9] Li H., Pan S.J., Wang S., Kot A.C. Domain generalization with adversarial feature learning. Proceedings of the IEEE Conference on Computer Vision and Pattern Recognition.

[10] Orgo L., Bachmann M., Kalev K., Jarvelaid M., Raik J., Hinrikus H. (2017). Resting EEG functional connectivity and graph theoretical measures for discrimination of depression. Proceedings of the 2017 IEEE EMBS International Conference on Biomedical & Health Informatics (BHI).

[11] Thoduparambil P.P., Dominic A., Varghese S.M. (2020). EEG-based deep learning model for the automatic detection of clinical depression. Phys. Eng. Sci. Med..

[12] Deng X., Fan X., Lv X., Sun K. (2022). SparNet: A convolutional neural network for EEG space-frequency feature learning and depression discrimination. Front. Neuroinform..

[13] Cai H., Yuan Z., Gao Y., Sun S., Li N., Tian F., Xiao H., Li J., Yang Z., Li X. (2022). A multi-modal open dataset for mental-disorder analysis. Sci. Data.

[14] Wang B., Kang Y., Huo D., Chen D., Song W., Zhang F. (2023). Depression signal correlation identification from different EEG channels based on CNN feature extraction. Psychiatry Res. Neuroinform..

[15] Seal A., Bajpai R., Agnihotri J., Yazidi A., Herrera-Viedma E., Krejcar O. (2021). DeprNet: A deep convolution neural network framework for detecting depression using EEG. IEEE Trans. Instrum. Meas..

[16] Chen T., Guo Y., Hao S., Hong R. (2022). Exploring self-attention graph pooling with EEG-based topological structure and soft label for depression detection. IEEE Trans. Affect. Comput..

[17] Li W., Wang H., Zhuang L. (2023). GCNs–FSMI: EEG recognition of mental illness based on fine-grained signal features and graph mutual information maximization. Expert Syst. Appl..

[18] Zhang Z., Meng Q., Jin L., Wang H., Hou H. (2024). A novel EEG-based graph convolution network for depression detection: Incorporating secondary subject partitioning and attention mechanism. Expert Syst. Appl..

[19] Liu W., Jia K., Wang Z. (2024). Graph-based EEG approach for depression prediction: Integrating time-frequency complexity and spatial topology. Front. Neurosci..

[20] Navarro-Bravo B., Latorre J.M., Jiménez A., Cabello R., Fernández-Berrocal P. (2019). Ability emotional intelligence in young people and older adults with and without depressive symptoms, considering gender and educational level. PeerJ.

[21] Montemurro S., Borek D., Marinazzo D., Zago S., Masina F., Napoli E., Filippini N., Arcara G. (2024). Aperiodic component of EEG power spectrum and cognitive performance are modulated by education in aging. Sci. Rep..

[22] Van Putten M.J.A.M., Olbrich S., Arns M. (2018). Predicting sex from brain rhythms with deep learning. Sci. Rep..

[23] Zhang X., Li J., Hou K., Hu B., Shen J., Pan J. (2020). EEG-based depression detection using convolutional neural network with demographic attention mechanism. Proceedings of the 2020 42nd Annual International Conference of the IEEE Engineering in Medicine & Biology Society (EMBC).

[24] Ksibi A., Zakariah M., Menzli L.J., Saidani O., Almuqren L., Hanafieh R.A.M. (2023). Electroencephalography-based depression detection using multiple machine learning techniques. Diagnostics.

[25] Sun S., Li J., Chen H., Gong T., Li X., Hu B. (2020). A study of resting-state EEG biomarkers for depression recognition. arXiv.

[26] Jas M., Engemann D.A., Bekhti Y., Raimondo F., Gramfort A. (2017). Autoreject: Automated artifact rejection for MEG and EEG data. NeuroImage.

[27] Vaswani A., Shazeer N., Parmar N., Uszkoreit J., Jones L., Gomez A.N., Kaiser Ł., Polosukhin I. (2017). Attention is all you need. Adv. Neural Inf. Process. Syst..

[28] Altaheri H., Muhammad G., Alsulaiman M. (2022). Physics-informed attention temporal convolutional network for EEG-based motor imagery classification. IEEE Trans. Ind. Inform..

[29] Bai S., Kolter J.Z., Koltun V. (2018). An empirical evaluation of generic convolutional and recurrent networks for sequence modeling. arXiv.

[30] Lu N., Yin T., Jing X. (2020). Deep learning solutions for motor imagery classification: A comparison study. Proceedings of the 2020 8th International Winter Conference on Brain-Computer Interface (BCI).

[31] Ingolfsson T.M., Hersche M., Wang X., Kobayashi N., Cavigelli L., Benini L. (2020). EEG-TCNet: An accurate temporal convolutional network for embedded motor-imagery brain–machine interfaces. Proceedings of the 2020 IEEE International Conference on Systems, Man, and Cybernetics (SMC).

[32] Duc T.N., Minh C.T., Xuan T.P., Kamioka E. (2020). Convolutional neural networks for continuous QoE prediction in video streaming services. IEEE Access.

[33] Liu C., Wu H., Cheng G., Zhou H., Pang Y. (2025). Rolling Bearing Degradation Identification Method Based on Improved Monopulse Feature Extraction and 1D Dilated Residual Convolutional Neural Network. Sensors.

[34] Siddique M.F., Saleem F., Umar M., Kim C.H., Kim J.-M. (2025). A hybrid deep learning approach for bearing fault diagnosis using continuous wavelet transform and attention-enhanced spatiotemporal feature extraction. Sensors.

[35] Salk R.H., Hyde J.S., Abramson L.Y. (2017). Gender differences in depression in representative national samples: Meta-analyses of diagnoses and symptoms. Psychol. Bull..

[36] Wang Y., Zhang X., Li Y., Qin H., Li X. (2024). Gender differences in the prevalence, correlated factors and comorbidity of depression in adolescents: A cross-sectional study in Shanghai, China. Front. Public Health.

[37] Hasanzadeh F., Mohebbi M., Rostami R. (2020). Graph theory analysis of directed functional brain networks in major depressive disorder based on EEG signal. J. Neural Eng..

[38] Sun S., Chen H., Shao X., Liu L., Li X., Hu B. (2020). EEG based depression recognition by combining functional brain network and traditional biomarkers. Proceedings of the 2020 IEEE International Conference on Bioinformatics and Biomedicine (BIBM).

[39] Mumtaz W., Xia L., Yasin M.A.M., Ali S.S.A., Malik A.S. (2017). A wavelet-based technique to predict treatment outcome for major depressive disorder. PLoS ONE.

